# The Effect of Tamsulosin in the Medical Treatment of Distal Ureteral Stones

**DOI:** 10.5539/gjhs.v6n7p44

**Published:** 2014-09-18

**Authors:** M. Alizadeh, M. Magsudi

**Affiliations:** 1 Nephrology and kidney Transplant Research Center, Urmia University of Medical Sciences, Urmia, Iran; 2 Urmia University of Medical Sciences, Urmia, Iran

**Keywords:** Distal ureteral stones, Indomethacin, Tamsulosin, Ureteral, Ureteroscopy

## Abstract

**Introduction:**

Renal stones are common disorders that affect approximately 5% to 10% of the population and the incidence of renal stones is rising. Treatment of ureteral stones is an important part of urologists and minimally invasive procedures such as ESWL and ureteroscopy effectiveness has been proven in various studies. However, these methods are not completely safe and are expensive and can be complicated. Purpose of this study is to evaluate the effectiveness of tamsulosin in the medical treatment of distal ureteral stones.

**Patients and methods:**

A total of 96 patients with distal ureteral stones or UVj are randomly divided into two study group (50 patients) and control group (46 patients). Patients in the control group allowed to freely consuming fluids (hydration) and indomethacin 100 mg PRN. Study group in addition to indomethacin and daily analgesic 0.4 mg tamsulosin was administered. All subjects in terms of analgesic dose, duration of expulsion and expulsion were studied.

**Results:**

Spontaneous expulsion of stone was occurred in 62.5% (30 patients out of 46) of control group patients and 82% (41 patients out of 50) that there was no significant difference (P>0.05). Average time to fix the stone in control group 4.7±8.03 days (range 2 to 28 days) and in the study group, 3.7±5.70 days (range 1 to 23 days) is significantly different (P>0.05). The average amount of analgesic consumption in the control group was 2.3±4.31and in the study group was 1.48±2.15 that showed significant differences (P<0.05).

**Conclusions:**

In this study, although the addition of tamsulosin to conservative treatment of distal ureteral stones in the distal ureteral stone expulsion showed no significant difference between the two groups, but the reduction in the duration of expulsion, reduce pain and reduce the need for analgesic has been beneficial.

## 1. Introduction

Renal stones are common disorders that affect approximately 5% to 10% of the population and of 100,000 outpatients visit in the US 122 visits are related to the patients with renal stones and the incidence of renal stones is rising (Pak, 1998; Hiatt et al., 1982). Treatment of ureteral stones is an important part of urologists and minimally invasive procedures such as ESWL and ureteroscopy effectiveness has been proven in various studies (Sierakowski et al., 1978; Carstensen & Hansen, 1973; Miller & Kane, 1999; Segura et al., 1997). However, these methods are not completely safe and are expensive and can be complicated (Lotan, 2002). On the other hand the expected treatment can be used in large numbers of patients. In different studies it is shown that small ureteral distal stones up to 98% may be excreted with expectant treatment and patient recovery (Hubner et al., 1993; Ueno et al., 1977; Coil et al., 2002). However, expected treatment can lead to complications such as urinary tract infections - hydronephrosis or negative effect on renal function (Ueno et al., 1977). So the choice between minimally invasive therapies and expected treatment, especially in patients with less symptoms or small urethral stones, is difficult (Moradi et al., 2010). Recently, expected treatment with pharmacological treatments that reduce the symptoms and accelerate the expulsion is expanding (Borghi et al., 1994; Poppiglia et al., 2000; Cervenakov et al., 2002; Morita et al., 1987). A number of researchers have published reports stating stone expulsion using Alpha-blocker (Tamsulosin) (Cervenakov et al., 2002; Morita et al., 1987). Their reason is that the alpha - 1 receptors have an important role in the physiology of the distal ureter (Morita et al., 1987; Malin et al., 1970; Morse & Resnick, 1991). It has been shown that medical treatment for ureteral stones in the management and following-up of patients with distal ureteral stones is helpful. Alpha-1 adrenergic receptor antagonists are being studied a lot in this field and has been reported that they can be useful in the treatment of ureteral stones (Pak, 1998). There are abundant Alpha-1 adrenergic receptors in the distal ureter and it is stated that Alpha-blocker is effective in the passage and expulsion of distal ureteral stone and reduce the duration of expulsion and reduce the need to take pain medication until expulsion (Hiatt et al., 1982).

It is reported that Alpha-blocker accelerates the expulsion of crushed stones after shock wave lithotripsy (SWL) and alleviate symptoms associated with ureteral stent and increase the tolerance of ureteral stent in allergic patients and perhaps in the true selection of patients, the use of Alpha-blocker be useful in the management of patients with distal ureteral stones (Sierakowski et al., 1978). Recent advances in endoscopic management of renal stones have allowed renal stones by minimally invasive techniques are successfully treated. These improvements include shock wave lithotripsy, ureteroscopy (TUL) and percutaneous nephrostolithotomy. Although these treatments are less invasive than traditional surgical methods, these methods are more expensive and have side effects related to themselves. Observations in the treatment of small ureteral stones, which is probable to expulsion and yet had not absolute indications for surgical intervention, are recommended. Ureteral stone expulsion rate 5mm and smaller in the proximal with no medical intervention is estimated to be 29% to 98%. And the most important factor in predicting passage and spontaneous expulsion of renal stones include placement of stones in the ureter and the largess of ureteral stones (Morita et al., 1987). Recently, many studies about medical expulsion therapy (MET) of ureteral stones with pharmacologic materials are being conducted that indicate the increasing possibility of spontaneous expulsion of renal stones (which are not predictable). Since edema and spasm of the ureter can have inappropriate effect on the passage of ureteral stone to remove the spasm, and ureteral edema the pharmacologic agents are being used and with this aim materials such as calcium channel blockers, steroids and non-steroid anti-inflammatory drugs and the alpha-1-adrenergic receptor antagonist have been used for MET of ureteral stones. A recent meta-analysis study is conducted in which compare the expulsion of ureteral stone with two alpha-1-adrenergic receptor antagonist and calcium channel blocker drugs and showed that prescription of any of these medications to patients with ureteral stones increase 65% the chance of expulsion (Hollingsworth et al., 2006). So we have decided to use a study comparing expected treatment with tamsulosin and expected treatment without using this Alpha-blocker (Tamsulosin) factors and evaluate pharmacological factors in distal ureteral stone expulsion.

## 2. Methods

In this randomized clinical trial, 102 patients aged 18 to 60 years from June 2007 until July 2008 due to renal colic (3 to 6 mm ureteral stone of distal ureteral or UVj) were referred to Clinic of Urology, Radiology Center or emergency of Urmia Imam Khomeini Hospital and after history and examination of public health (absence of hypertension or high blood pressure medicines - hypotension (systolic pressure less than 100) - lack of surgical ureteral stones - no history of diabetes - lack of peptic ulcers or intolerance to NSAIDs - non-calcium antagonist drugs such as nifedipine and no history of renal disease and pregnancy tests on married women) were selected for the project. Of these 6 patients due to non-cooperation in different stages of study (in on time reference or regular drug use or lack of access to patient) were excluded and finally 96 patients were remained in the study. Exclusion criteria are included:

1 - UTI
2 - Radiolucent stones on KUB
3 – Acute hydronephrosis (grades 2 and 3) in sonography
4 - Diabetes (FBS greater than 125 mg per deciliter)
5 - Patients with a history of peptic ulcer disease
6 - Systolic blood pressure less than 100
7 - Consumers of calcium antagonist drugs
8 - Patients with a history of surgery on the distal ureter
9 - The single renal patients
10 - Creatinine over 1.4 for males and 1.2 in females
11 - Pain resistant to conservative treatment (non-tolerant patients)
12 - Patients with NSAID drug intolerance or adverse effects of Tamsulosin during study
13 - The patient withdrew from the study at any time.
14 - The occurrence of any unforeseen complications (NSAID drug intolerance or tamsulosin) during the study
15 - Pregnancy

Patients before enrolling to the study were examined and investigated medically and how do the study and treatment of distal ureteral stones was explained to them. All patients before study were examined and investigated with KUB and sonography of the urinary system, and the size and location of the stone and the degree of renal hydronephrosis were recorded and for patients CBC, FBS, BUN, Cr, UA, UC requested, and patients were randomly assigned to a control group (n = 46) and study group (n = 50). The study group was given daily tamsulosin 0.4mg. Tamsulosin was administered during the morning, until the prescribed period of expulsion or non-expulsion for up to four weeks after the beginning of the study. All of the patients were allowed to use symptomatic treatment indomethacin 100mg as PRN (unbearable pain) and all patients from each group were emphasized to drink two liters of water daily. Stone expulsion, pain episodes and analgesic dose were investigated and of all patients every two weeks with KUB, UA, UC and sonography in terms of ureteral stone expulsion and possible side effects resulting from ureteral stone were studied and in case of side effects recorded in the patients’ medical records and patients who had distal ureteral stone expulsion after 4 weeks were introduced for TUL.

The data in terms of

1 - The size and location of ureteral stones
2 - Degree of hydronephrosis
3 - The rate of expulsion
4 - Time of expulsion
5 - The amount of analgesic consumption
6 - Possible complications caused by stones during the study period
7 - Possible side effects from medications or intolerance recorded and used for statistical analysis.

For data analysis of the present study, statistical description include frequency tables, graphs, percentage and mean and standard deviation and then statistical inference based on parametric methods (T-test and the variance) and non-parametric (Chi-square) were achieved. In analyzing the data, the statistical error at 0.05 was considered significant. And SPSS 16 and Exell 2003 software were used for data analysis (Asli et al., 2013).

## 3. Results

In this research, the study group, 50 patients (21 female and 29 male, age limit 20-50 years) and control group, 46 patients (14 females and 32 males, age limit 19-54 years) were selected. In Figure 1 the frequency of male and female in the two groups are drawn. The mean stone size of the study group and control group is 0.93±4.6 and 0.83±4 .81, respectively. The mean stone size between male and female in the two groups is shown in Figure 2. Age and size of the stones in the two groups shower no significant difference (P>0.05). During the study period no symptoms (such as UTI, fever, severe obstructive uropathy, worsening of symptoms and side effects of tamsulosin or indomethacin that require discontinuation of medication) was observed. Table 1 shows the locations of stones and their distribution in the study and control groups. Spontaneous expulsion of stone was occurred in 62.5% (30 patients out of 46) of control group patients and 82% (41 patients out of 50) that there was no significant difference (P>0.05). In Figure 3, the frequency of expulsion in control and study groups was drawn. The mean expulsion time in the control group 4.7±8.03 days (range 2 to 28 days) in the study group, 3.7±5.07 days (range 1 to 23 days) that is significantly different (P<0.05). When the size of the stone expulsion in the two groups were compared, no significant difference was observed (P>0.05). The average amount of analgesic consumption in the control group was 2.3±4.31 (1 to 10) and in the study group was 1.48±2.15 (0 to 5) which had significant differences (P<0.05).

**Table 1:** Stone location, frequency and distribution percentage in the two groups

Group	L distal	L uvj	R distal	R uvj	Total
Control	19(42.2%)	8(17.8%)	8(17.8%)	10(22.2)	45
Study	15(30%)	7(14%)	18(36%)	10(20%)	50
Total	34	15	26	20	95

## 4. Discussion

Articles express differing results in the ureteral stone expulsion (MET). Cervenakov and his group conducted the first double-blind randomized study to compare MET with and without tamsulosin. Standard treatment involves injecting a narcotic drug and diazepam at the time of their visit and then continues daily treatment with NSAID. They show that the stone expulsion with or without tamsulosin was 80.4% and 62.8% respectively. In their study, most of the patients receiving tamsulosin had ureteral stone expulsion within 3 days and their pain was of control group. They concluded that tamsulosin increased the stone expulsion rate and decreased expulsion time. In addition, the drug showed a reduction in pain during the time of expulsion (Cervenakov et al., 2002). Dellabella et al. 60 patients with symptomatic stones UVj in two groups of 30 subjects that in the study group tamsulosin and in the control group Floroglucinetrimetossibenzene (a spasmolytic drug used in Italy) was prescribed. In addition they prescribed to all the patients in both groups, an oral steroid (deflazacot) for 10 days and diclofenac as PRN. Expulsion for the group receiving tamsulosin was 100% compared to 70% for the control group. Tamsulosin group expulsion time was significantly lower, 65.7 hours in the control group versus 111.1 hours in the study group. In addition, the group receiving tamsulosin had less need of diclofenac injection (0.13 units compared to 2.83 units) (Dellabella et al., 2003). Resim et al. investigated on 60 patients with distal ureteral stones that were divided into control group (receiving tenoxica and an NSAID) and study group (receiving tenoxica and tamsulosin). Stone size in study group was 5 to 12 mm and in the group receiving tamsulosin was 5 to 13 mm. The stone expulsion rate was 86.6% in the group receiving tamsulosin compared with 73.3% in the control group and none of the patients required discontinuation due to side effects of tamsulosin or not excluded. In addition, they showed that the group receiving tamsulosin had significantly less pain (based on a 10-part grading 5.70 vs 8.30, P <.0001 VAS visual analogue scale) and experienced less renal colic (De Sio et al., 2006). In the present study stone expulsion was occurred in 62.5% (30 out of 46) of the control group and 82% (41 out of 50) of the study group that had no significant difference. However, the mean expulsion time was significantly lower in the group receiving tamsulosin than control group that confirmed some of the results of previous studies. In addition, analgesics mean in the study group compared with the control group showed a significant decrease that is consistent with the above studies. Therefore, according to the present study and the mentioned studies using tamsulosin is appropriate in patients with distal ureteral stones that have no infection, unbearable pain, renal failure and contraindications of the drug to accelerate the stone expulsion, reduce the time of expulsion and decrease analgesic use.

## 5. Conclusions

In this study, although the addition of tamsulosin to conservative treatment of distal ureteral stones in the distal ureteral stone expulsion showed no significant difference between the two groups, however, was effective in decreasing expulsion time, decreasing pain, and also reducing the need for analgesics. We suggest that future studies should be designed with a larger sample size to investigate the probability of the effect of tamsulosin on ureteral stone expulsion rate.
